# Légionellose compliquée d'une rhabdomyolyse et d'une insuffisance rénale aiguë: à propos d'un cas

**DOI:** 10.11604/pamj.2016.24.126.8536

**Published:** 2016-06-09

**Authors:** Arnaud Bac, Ahmed Sabry Ramadan, Pierre Youatou, Pierre Mols, Dominique Cerf, William Ngatchou

**Affiliations:** 1 Service des Urgences du CHU St Pierre de Bruxelles, Université libre de Bruxelles, Belgique; 2 Service de Chirurgie Cardiaque et des Urgences CHU St Pierre de Bruxelles, Université Libre de Bruxelles, Belgique

**Keywords:** Legionnaires´ disease, rhabdomyolysis, renal failure, Legionnaires' disease, rhabdomyolysis, renal failure

## Abstract

La légionellose est une maladie respiratoire bactérienne due à un germe gram négatif dont la présentation clinique peut être bénigne se limitant à un syndrome grippal ou plus sévère se caractérisant par une pneumonie pouvant se compliquer d'atteinte multisystémique pouvant conduire au décès. Nous rapportons le cas d'un patient de 48 ans ayant présenté une rhabdomyolyse compliquée d'une insuffisance rénale aigue au décours d'une pneumonie à Legionella pneumophila. Nous revoyons la physiopathologie et le traitement de cette complication rare de la légionellose.

## Abstract (en)

Legionnaires’ disease is a bacterial disease of the respiratory system caused by a gram-negative germ whose clinical manifestation can be benign limiting to flu-like syndrome or can be more severe being characterized by pneumonia which may be complicated by multisystem disease that can lead to death. We report the case of a 48 year-old patient with rhabdomyolysis complicated by acute renal failure following Legionella pneumophila pneumonia. We here highlight the pathophysiological aspects and treatment of this rare complication during Legionella infection.

## Introduction

La légionellose est une maladie infectieuse bactérienne, causée par un bacille à gram négatif, dont il existe plusieurs espèces et sérogroupes [1]. Legionella pneumophila est le plus souvent mise en cause et tout particulièrement le sérogroupe I (Lp1), connu depuis l’épidémie de 1976 lors d'un congrès de l'American Légion [1, 2]. Il s'agit d'une bactérie ubiquitaire, exigeante, à développement intracellulaire, qui a pour habitat le plus répandu les eaux douces naturelles et les sols humides [1–3]. La légionellose est une maladie à déclaration obligatoire dans plusieurs pays européens mais souvent sous-déclarée car elle est le plus souvent sporadique plutôt qu’épidémique [1, 2]. La contamination se fait par l'inhalation de gouttelettes d'eau infectée par le germe présent dans les systèmes de canalisation d'eau ou de climatisation [1–3]. Les tours refroidissantes, les saunas, les bains à remous, les fontaines, les salles de bains, les respirateurs, les nébulisateurs sont connus comme des sources d'infection à Legionella [1–3]. La légionellose se manifeste progressivement sous deux tableaux: la forme sporadique ou fièvre de Pontiac qui ressemble à un syndrome grippal, la forme plus sévère qui est pneumonie pouvant conduire à une faillite multisystémique et au décès [1–5]. La rhabdomyolyse est complication rare de la légionellose dont le mécanisme reste inconnu [6, 7]. Cette présentation sévère est signalée dans environ 15% de cas; ses complications induisant un taux de mortalité de plus de 50% pouvant s’élever à 80% en fonction du terrain de l'hôte [6, 7].

## Patient et observation

Un patient de 48 ans originaire de l'Afrique subsaharienne se présente aux urgences avec un tableau de douleur abdominale diffuse, de nausée, d'anorexie et de rectorragie sur fond de constipation débuté trois jours auparavant. Les rectorragies n'ont pas inquiété le malade car il a un antécédent d'hémorroïdes. Quelques heures avant son admission, le patient décrit une dégradation de son état général avec l'apparition d'une dyspnée modérée, de la fatigue et d'une myalgie généralisée. L'anamnèse ne relève pas de vomissement ni de plainte urinaire. L'examen clinique met en évidence une polypnée légère sans orthopnée, un teint grisâtre et des extrémités froides; mais avec un temps de recoloration capillaire inférieur à 2 secondes. La température corporelle est mesurée à 36.7°C. Le rythme cardiaque est mesuré à 104/min, la tension artérielle à 120/80 mmHg et la saturation à 98% à l'air ambiant. L'auscultation pulmonaire révèle de râles humides à la base droite, l'examen de l'abdomen montre une sensibilité généralisée sans défense ni rebound. Le péristaltisme est normal. La biologie sanguine à l'arrivée montre une hémoglobine à 17.9 g/dl, des globules blancs à 14830/µL, avec 81.4% de neutrophiles, une CRP à 119 mg/l ( N 1-10), une urée à 176 mg/dl (N 13-40) une créatinine à 9.69 mg/dl (N 0.55-0.96), une GRF à 6 ml/min (N >60), une Kaliémie à 5.9 mmol/L(N 3.45-4.45), un AST 1917 UI/L (N >32), un ALT 446 UI/L (N 6-34), un LDH 45766 UI/L (N 240-480) et des CPK à plus de 600.000 UI/L (N 1-172). Devant cette rhabdomyolyse importante et l'insuffisance rénale aiguée, une échographie abdominale, un CT scan abdominal sans injection et une radiographie de thorax sont réalisés et ne montrent pas d'anomalie. Des prélèvements bactériologiques comprenant deux paires d'hémocultures, des sérologies mycoplasme et chlamydiae pneumoniae ainsi qu'un examen microscopique des urines avec culture ont également été réalisés. Hormis la positivité de l'Ag légionnelle urinaire, le reste du bilan infectieux et auto-immunitaire revient négatif. Le patient sera admis aux soins intensifs. Durant son séjour dans cette unité, il développera une anurie persistante nécessitant une hémodialyse transitoire. Dix jours après son admission, la radiographie du thorax montrera l'apparition d'un foyer pulmonaire basal droit. Après 15 jours d'antibiothérapie et de dialyse itérative, on notera une amélioration clinique et la reprise progressive d'une diurèse permettant la sortie du patient des soins intensifs 17 jours après son admission. L'anamnèse rétrospective de ce patient ne révèle pas d'histoire de manipulation de chaudière de climatisation ou la fréquentation des bains publics les jours avant. Une déclaration au ministère de la Santé Publique sera faite pour une étude épidémiologique qui reviendra négative.

## Discussion

La rhabdomyolyse importante associée à une insuffisance rénale aiguë secondaire chez notre patient relève une des manifestations sévères et rare de légionellose [6, 7]. La sévérité clinique de cette maladie dépend de l'hôte, de la rapidité avec lequel le diagnostic est posé et l'antibiothérapie adéquate instaurée [4–7]. Bien que peu symptomatique sur le plan respiratoire aux urgences, notre patient était porteur d'une légionellose depuis quelques jours, malgré l'absence de facteurs de risque environnementaux [1–3].

### Diagnostic de la légionellose

La légionellose n'ayant pas de spécificité radioclinique, son diagnostic doit être évoqué devant toute pneumopathie avec [1]: un contexte nosocomial ou épidémique: voyage, thermes, exposition à de l'eau en aérosols; tableau clinique évocateur: pneumonie d'allure sévère, atteinte neurologique et/ou multisystémique; présence des signes biologiques évocateurs: cytolyse hépatique, syndrome glomérulaire et/ou insuffisance rénale, rhabdomyolyse; Échec préalable des bêtalactamines actives sur le pneumocoque; patients avec terrain favorisant (cancer, immudépression). La clinique atypique et peu symptomatique de notre patient sur le plan respiratoire, ainsi que l'absence des facteurs environnementaux nous poussent à diviser le diagnostic biologique en deux groupes.

La difficulté diagnostic restant comme pour notre patient les cas où le tableau pulmonaire reste en arrière plan. D'où l'intérêt de la recherche de l'antigène soluble urinaire, faite dans ce cas aux urgences de manière fortuite. Le Tableau 1 résume les méthodes pour confirmer le diagnostic de légionellose avec leurs avantages et inconvénients [1].

**Tableau 1:** Méthodes de diagnostiques

Méthodes	Sensibilité	Spécificité	Avantages	Inconvénients
Ag soluble urinaire	56-80%	99%	Rapides +++même sous traitement Diagnostic et Traitement précoces A permis de diminuer la mortalité	Ciblé sur le L.P 1 Cher En routine? Risque de sous diagnostiquer les autres sérotypes
Culture	60%	100%	Gold standard Toutes espèces 3-5j	Milieux spéciaux Négativation rapide et peu sensible sous traitement Demande Spécifique
Sérologie	80%	97-99%	épidémiologique	Peu d'intérêt en aigue
Immunofluorescence directe	25%	65%	Rapide	Labos spécialisées Réactions croisées

### Traitement de la légionellose

Les antibiotiques avec une pénétration intracellulaire constituent la base du traitement de la légionellose. Parmi ceux-ci, on retrouve les macrolides, les quinolones, les tétracyclines et la rifampicine [8, 9]. Les bêtalactamines sont totalement inactives. L’érythromycine n'est plus l'antibiotique de 1^er^ choix, la télithromycine ne fait pas mieux que l’érythromycine, les quinolones sont supérieures à l’érythromycine et aux macrolides en général sauf l'azithromycine [8]. La durée de traitement est de 8 à 14 jours pour les formes légères et de 14 à 21 jours avec admission aux soins intensif pour les formes sévères [6–9].

### Pronostic de la légionellose

Le pronostic de la légionellose dépend de l'hôte, de la précocité du diagnostic et de la rapidité d'instauration d'un traitement adapté ainsi que de la forme de la maladie [8]. Environ 15% des cas se présentent sous la forme sévère, avec un taux de mortalité de plus de 50%, pouvant s’élever jusqu’à 80% en fonction du terrain de l'hôte [6, 7, 10].

### Rhabdomyolyse et légionellose

Les symptômes et signes cliniques de la rhabdomyolyse sont très variés [6, 7, 11, 12]. Chez notre patient on retrouve une asthénie et une myalgie généralisée. L'origine infectieuse est retenue en l'absence d'autres causes telles que l'origine traumatique, toxique ou médicamenteuse [11, 12]. Le diagnostic est posé sur le plan biologique par une valeur des CPK multipliée par 1000 [7, 11, 12]. Plusieurs hypothèses sont avancées pour expliquer la lyse musculaire dans les infections [7]: la fièvre et l’état de choc; La présence des particules microbiennes dans les fibres musculaires entrainant la production locale de prostaglandines E2; L'action des endotoxines circulantes sur le muscle. L'insuffisance rénale aigue généralement secondaire à la rhabdomyolyse évoluant comme chez notre patient vers l'oligurie voir l'anurie. Le traitement de la rhabdomyolyse dans ce contexte se fera par l'administration d'une antibiothérapie adéquate contre l'agent responsable et en un traitement symptomatique basé sur la réhydratation parentérale et des séances de dialyse.

## Conclusion

La pneumonie à légionnelle est une maladie communautaire, sévère et parfois mortelle. Les moyens diagnostiques rapides tels que la recherche de l'antigène urinaire soluble et l'imagerie ont permis une prise en charge plus rapide et une amélioration de son diagnostic. Cependant, les cliniciens doivent rester vigilants dans des cas avec présentation frustre où le tableau pulmonaire reste en arrière plan.
